# Supplementary figures and images for: Emergence of belief-like representations through reinforcement learning
Source: PLoS Comput Biol. 2023 Sep 11;19(9):e1011067. doi: 10.1371/journal.pcbi.1011067 (PMC10513382; doi:10.1371/journal.pcbi.1011067)

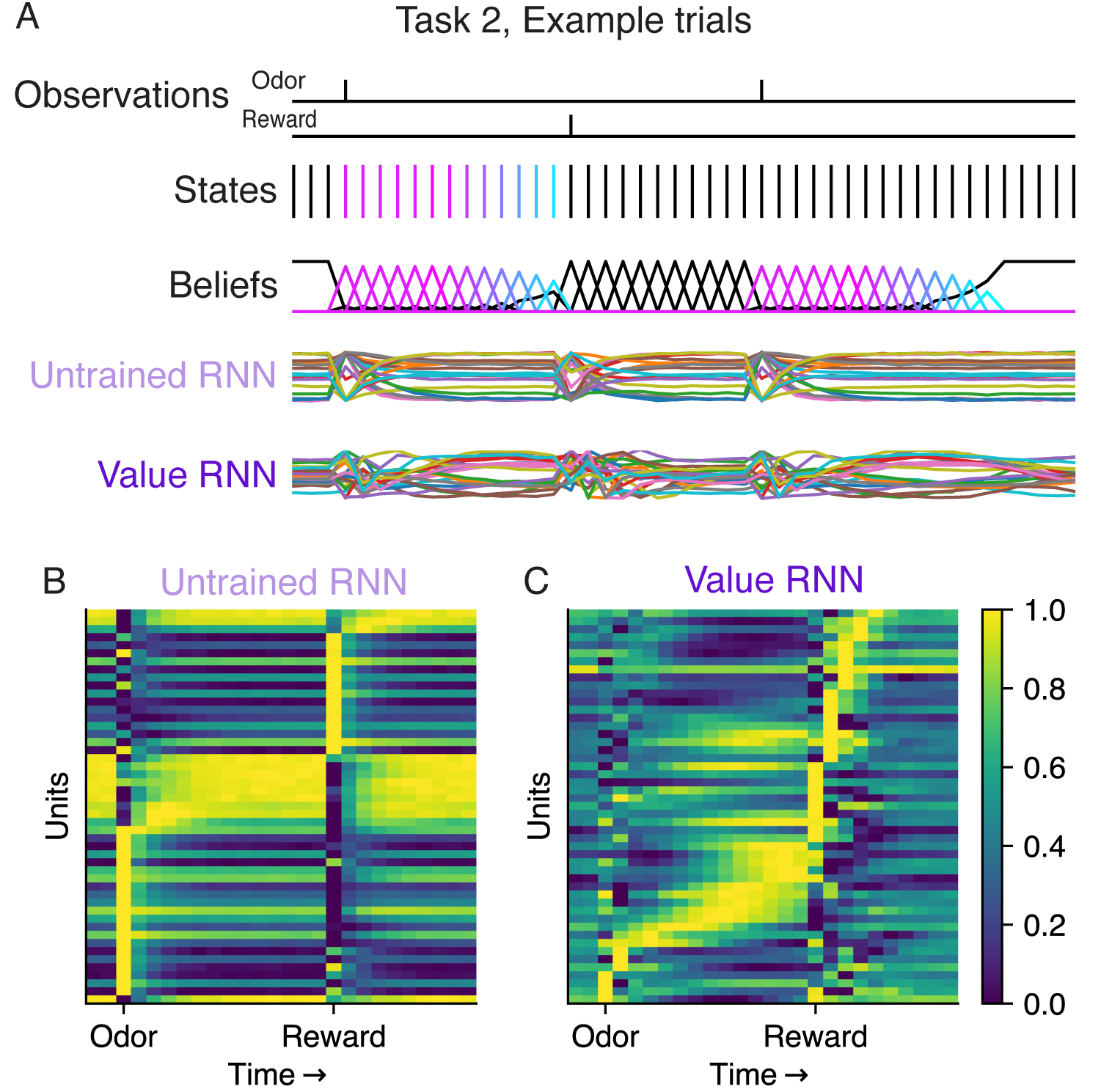

Supplement: S1 Fig — A. Example observations, states, beliefs, and Value RNN activity from the same Task 2 trials shown in Figs 2 and 4A. States and beliefs are colored as in Fig 2, with black indicating ITI microstates, and other colors indicating ISI microstates. B-C. RNN unit activity (individually normalized to span between 0 and 1), with units sorted by time of peak activation on held-out trials, on an RNN before (panel B) and after (panel C) training. Both before and after after training, RNN units exhibited tuning to elapsed time following observations, with variance that scaled with elapsed time. (TIF) [file pcbi.1011067.s001.tif]

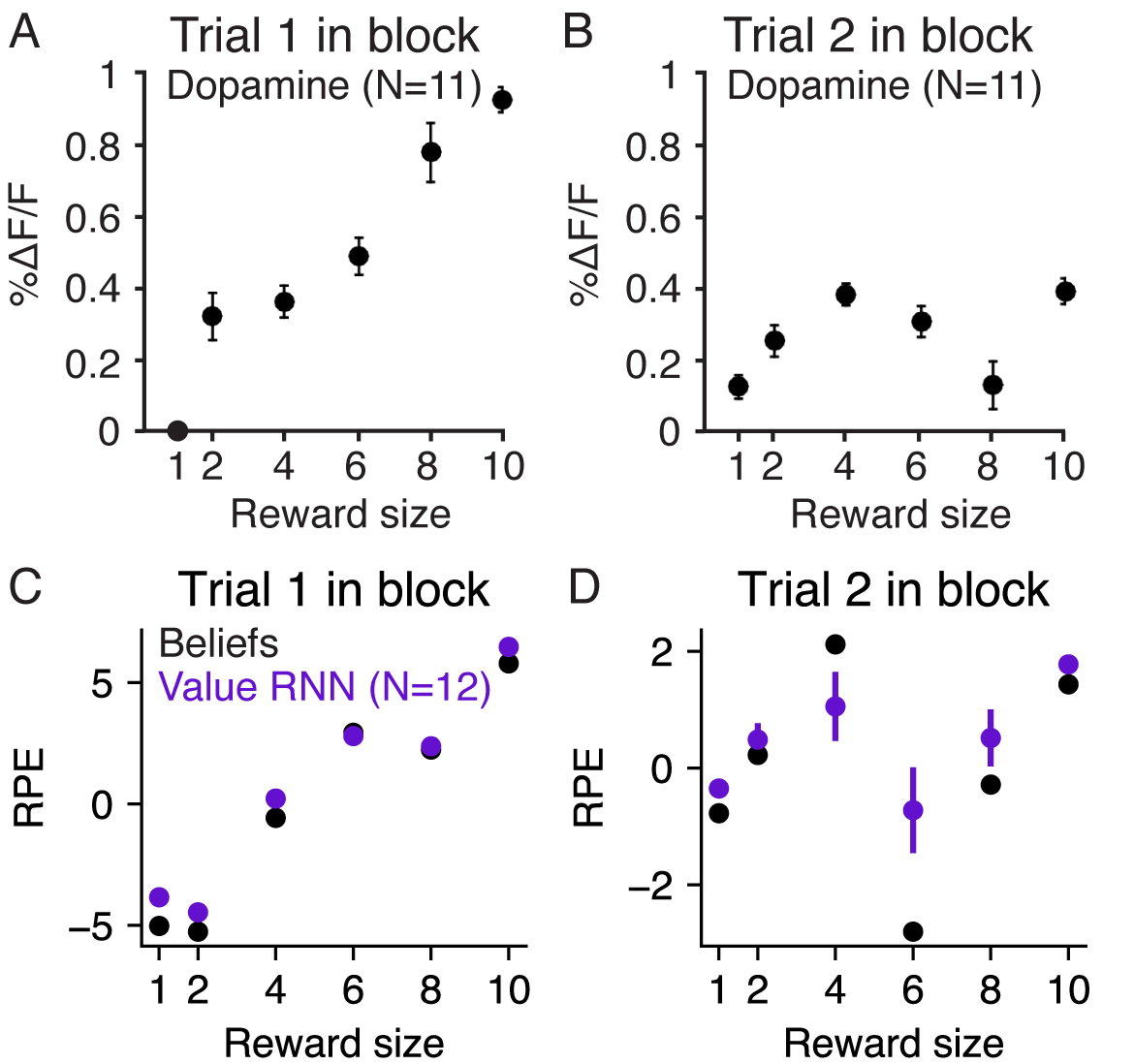

Supplement: S2 Fig — A-B. Average dopamine response on trial 1 (panel A) and trial 2 (panel B) during probe sessions including blocks with intermediate reward sizes. Circles and lines depict mean ± SE across N = 11 animals. Reproduced from Babayan et al. (2018) [10]. C-D. Same as panels A- B, but for the RPEs of the Belief model (black) and Value RNNs (purple). Value RNNs were trained on sessions including only blocks with rewards rt ∈ {1, 10}, as in the main text. Value weights for the Belief model and Value RNNs were fit using a test session including 39 blocks each with rt = 1 and rt = 10, and 3 blocks each with rt ∈ {2, 4, 6, 8}, similar to the proportions used in Babayan et al. (2018) [10]. RPEs were then measured on a different test session. Purple circles and lines depict mean ± SE across N = 12 models. (TIF) [file pcbi.1011067.s002.tif]

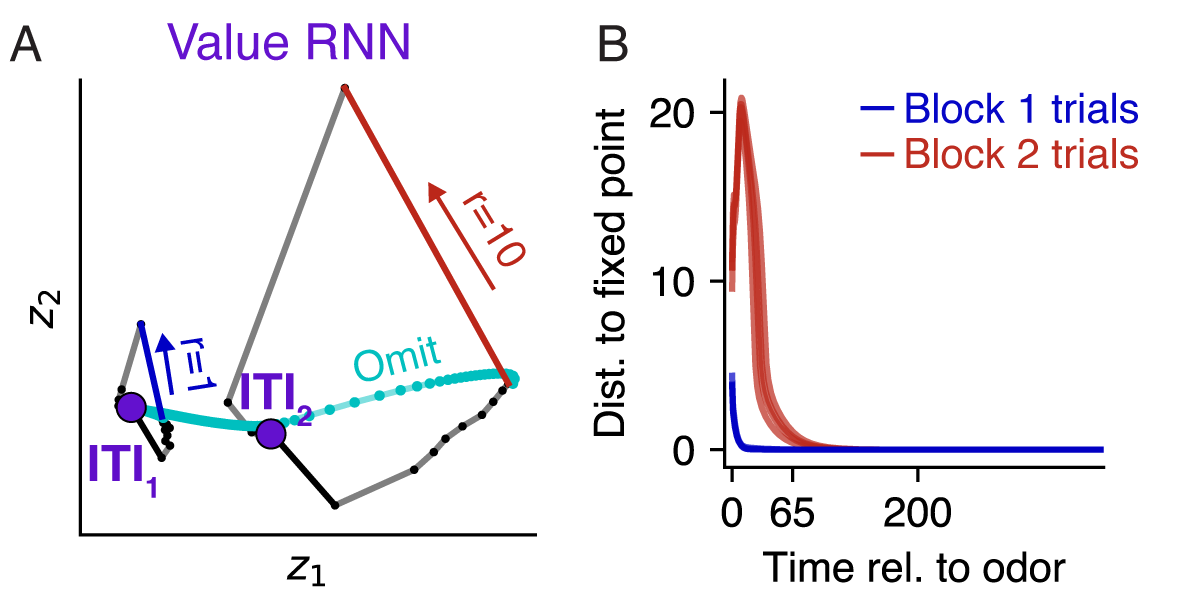

Supplement: S3 Fig — A. RNN activity during two example trials, one during Block 1 (left) and the other during Block 2 (right). Same as Fig 7D. Here we also include RNN activity trajectories if each reward had been omitted. While activity for the Block 2 trial initially returns to the putative ITI2 state, it eventually returns to the true fixed point at ITI1 B. Distance of RNN activity from the single fixed point (e.g., ITI1 in panel A) following an odor observation (i.e., an omission trial). While the maximum ITI duration is theoretically infinite, the maximum ITI duration in the training data was at t = 65. RNN activity on Block 2 trials therefore remained separate from the activity on Block 1 trials for the range of experienced ITI durations. (TIF) [file pcbi.1011067.s003.tif]

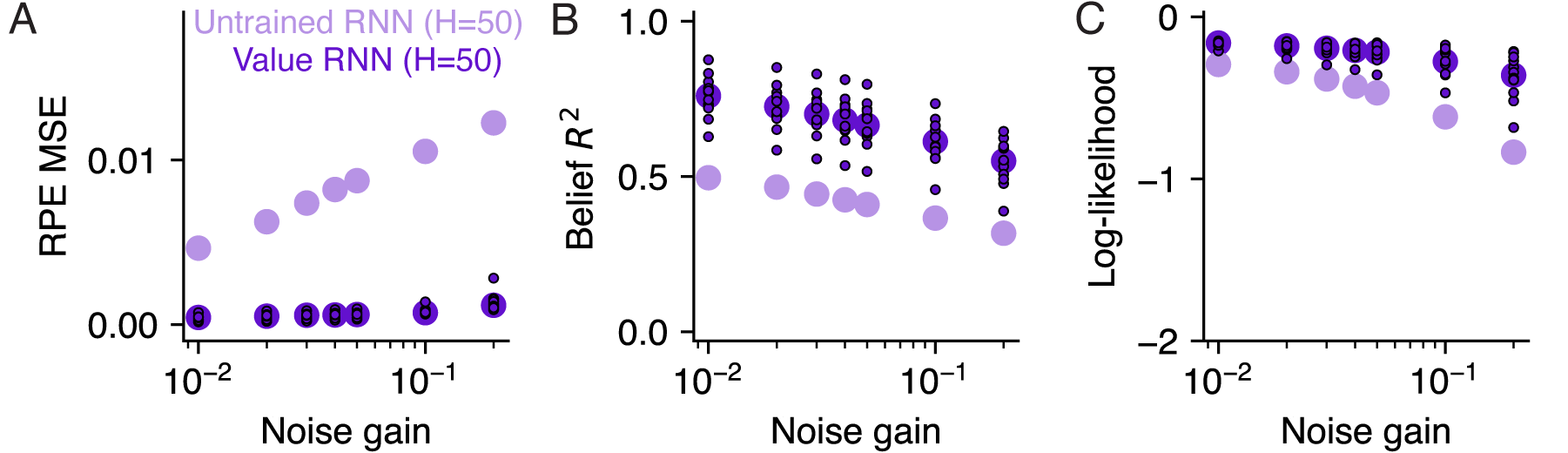

Supplement: S4 Fig — A. Error between the RPEs of the Value RNN (dark purple) and Untrained RNN (light purple) relative to the Belief model’s RPEs (“RPE MSE”; see Fig 3D) during Starkweather Task 2, as a function of the magnitude of the Gaussian noise added to each unit prior to analysis (see Materials and methods). All RNNs had 50 hidden units. Each dot is the error for a single Value RNN model. Each circle is the median across the N = 12 Value RNNs (dark purple) or N = 12 Untrained RNNs (light purple) at a given noise level. B. Total variance explained (R2) of beliefs on held-out trials (see Fig 4B). Same conventions as panel A. C. Cross-validated log-likelihood of the state decoder using RNN activity to estimate the true state (see Fig 4C). Same conventions as panel A. (TIF) [file pcbi.1011067.s004.tif]
